# Francis Peyton Rous

**DOI:** 10.3201/eid1904.130049

**Published:** 2013-04

**Authors:** Prasanna Kumar, Frederick A. Murphy

**Affiliations:** Mill Creek High School, Hoschton, Georgia, USA (P. Kumar);; University of Texas Medical Branch, Galveston, Texas, USA (F.A. Murphy)

**Keywords:** viruses, virology, cancer, oncology, sarcoma, malignancy, tumor-inducing factor, chickens, history, viral oncology

This is a photograph of Peyton Rous (Francis Peyton Rous, 1879–1970), who in 1909–11 made 2 seminal discoveries that are now the foundation blocks of modern virology and oncology. First, he discovered that a malignant tumor (a sarcoma in chickens) was transmissible; this was the first transmissible solid tumor discovered. Second, he found that the tumor-inducing factor could be passed through a Berkefeld ultrafilter known to retain bacteria. In the context of the times, this finding proved that the agent was a virus, the first of its kind. The virus he discovered is now known as Rous sarcoma virus and has since been studied in many laboratories around the world.

Rous was born in 1879 in Baltimore, Maryland, USA, and raised by his widowed mother, who persevered in supporting his education. He received bachelor of arts and doctoral degrees from The Johns Hopkins University in Baltimore in 1900 and 1905, respectively. After teaching pathology at the University of Michigan, Ann Arbor, and studying morbid anatomy at Friedrichstadt Municipal Hospital in Dresden, Germany, in 1909, he took a position at the Rockefeller Institute for Medical Research in New York, New York, where he spent the rest of his life. Rockefeller was the place in the United States where virology first emerged as a distinct medical science; from the beginning of his career, Rous was surrounded by great scientists.

Rous’ entry into tumor virology was fortuitous; the founding director of the Rockefeller Institute, Simon Flexner, had been interested in oncology but wanted to redirect his own work toward polio, which was becoming a major problem. Rous was hired to continue Flexner’s research into oncology, a subject about which Rous at first knew nothing.

The beginning of the story behind Rous’ claim to fame is remarkable: a woman came to the Rockefeller Institute with a barred Plymouth Rock hen that had a large tumor on its breast. Rous later wrote, “In this paper is reported the first avian tumor that has proved transplantable to other individuals. It is a spindle-celled sarcoma of a hen, which has thus far been propagated to the fourth generation….” In his research, he found that only closely related chickens were susceptible, but in these chickens, continuous passage of cell-free material led to tumors that grew quickly, were more malignant than usual, and produced widespread metastases. Rous continued to study the phenomenon he had begun to unravel for many years, but understanding the mechanistic bases for the complex natural history of Rous sarcoma virus and related viruses had to wait until modern molecular and cellular biologic technologies became available in the 1960s.

In 1934, Rous’ Rockefeller Institute colleague, Richard E. Shope, asked him to examine warts on jackrabbits that had definitively been shown to be caused by an ultrafilterable virus. This virus was Shope papillomavirus (rabbit papillomavirus). When Rous confirmed that the warts were benign tumors, he was reinvigorated in his intent to unravel the mysteries of viral oncology. Over the next 30 years, Rous and his colleagues showed that the benign tumors could progress to malignant carcinomas and that chemical carcinogens could interact with the virus—further discoveries that formed building blocks for modern virology. Today, we recognize that ≈20% of human cancers worldwide have infectious etiologies, for which preventive measures such as vaccines have great promise.

Rous’ colleagues, including the scientists René Dubos and Charles B. Huggins, lauded Rous’ personal and professional qualities, writing that he was gifted with supreme intellectual powers, unfailing integrity and honesty, a remarkably intuitive sense for the science itself, great perseverance and work ethic, and an enormous zest for life. One can imagine with wonder Rous in his laboratory and in the legendary lunchroom of the Rockefeller Institute.

Rous was duly honored for his masterful work, winning the National Medal of Science and membership in the National Academy of Sciences, the American Philosophical Society, the Royal Society, and other prestigious organization. In 1966, when he was 87 years old, Rous was awarded the Nobel Prize in Medicine, an honor he shared with Charles Huggins. After 55 years, the longest “incubation period” in the history of the Nobel Prizes, Rous’ discovery had finally been recognized with this honor. In the end, proof that Rous was just ahead of his time might be found in the several additional Nobel Prizes awarded since 1966 to virologists who further unraveled viral oncology.

**Figure:**
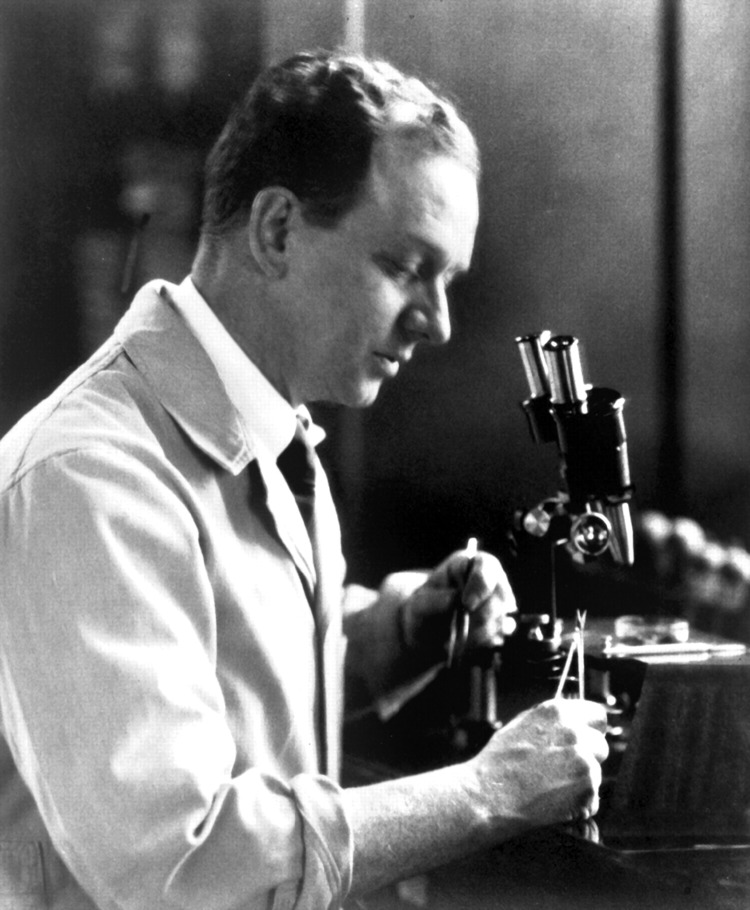
Francis Peyton Rous (1879–1970), pictured in 1923, at age 44, in his laboratory at the Rockefeller Institute for Medical Research, New York, NY, USA. Source: National Library of Medicine.
